# *MUG500+*: Database of 500 high-resolution healthy human skulls and 29 craniotomy skulls and implants

**DOI:** 10.1016/j.dib.2021.107524

**Published:** 2021-11-04

**Authors:** Jianning Li, Marcell Krall, Florian Trummer, Afaque Rafique Memon, Antonio Pepe, Christina Gsaxner, Yuan Jin, Xiaojun Chen, Hannes Deutschmann, Ulrike Zefferer, Ute Schäfer, Gord von Campe, Jan Egger

**Affiliations:** a Graz University of Technology (TU Graz), Graz, Styria, Austria; b Computer Algorithms for Medicine Laboratory (Café Lab), Graz, Styria, Austria; c Medical University of Graz (MedUni Graz), Graz, Styria, Austria; d Research Center for Connected Healthcare Big Data, ZhejiangLab, Hangzhou, Zhejiang, China; e Institute of Biomedical Manufacturing and Life Quality Engineering, School of Mechanical Engineering, Shanghai Jiao Tong University, Shanghai, China

**Keywords:** Skull, Cranial implant design, Craniotomy, Patient-specific implants (PSI), Computer-aided design (CAD), Machine learning, deep learning

## Abstract

In this article, we present a skull database containing 500 healthy skulls segmented from high-resolution head computed-tomography (CT) scans and 29 defective skulls segmented from craniotomy head CTs. Each healthy skull contains the complete anatomical structures of human skulls, including the cranial bones, facial bones and other subtle structures. For each craniotomy skull, a part of the cranial bone is missing, leaving a defect on the skull. The defects have various sizes, shapes and positions, depending on the specific pathological conditions of each patient. Along with each craniotomy skull, a cranial implant, which is designed manually by an expert and can fit with the defect, is provided. Considering the large volume of the healthy skull collection, the dataset can be used to study the geometry/shape variabilities of human skulls and create a robust statistical model of the shape of human skulls, which can be used for various tasks such as cranial implant design. The craniotomy collection can serve as an evaluation set for automatic cranial implant design algorithms.

## Specifications Table

**Table:** 

Subject	Information
Specific subject area	Computer Vision and Pattern Recognition
Type of data	Image
How data were acquired	The skulls are segmented from head computed tomography (CT) scans using a customized thresholding technique.
Data format	Raw
Parameters for data collection	The selection of DICOM files was based on the image quality (e.g., slice thickness, fracture, scanning protocol).
Description of data collection	The dataset includes two types of skulls: the 500 healthy skulls, each of which contains the complete bony structures of a human skull and the 29 craniotomy skulls, where a part of the cranial bone is missing on each skull.
Data source location	Medical University of Graz
Data accessibility	The download link of this dataset can be found from the Figshare repository^1^: https://figshare.com/s/e3d9debd55ad24c84678?file=17264471
Related research articles	jianning Li, Gord von Campe, Antonio Pepe, Christina Gsaxner, Enpeng Wang, Xiaojun Chen, UlrikeZefferer, Martin Tödtling, Marcell Krall, Hannes Deutschmann, et al. Automatic skull defect restoration andcranial implant generation for cranioplasty. Medical Image Analysis, 73:102171, 2021. DOI: https://doi.org/10.1016/j.media.2021.102171. reference: [1]

## Value of the Data

• The 500 healthy skulls can be used to create an statistical shape model (SSM) for cranial implant design [2], study the geometry variability of human skulls [3], [4], etc.
• The 29 craniotomy skulls together with the corresponding manually designed cranial implants can serve as an evaluation set for automatic cranial implant design algorithms.
• Researchers can create synthetic cranial defects on the 500 healthy skulls in order to train deep learning algorithms [1], [5], [6], [7] and host challenges [8].
• The *.stl* files included in the *MUG500+* dataset are 3D printable and can be used for educational purposes.

## 1 Data Description

**Fig. 1:**
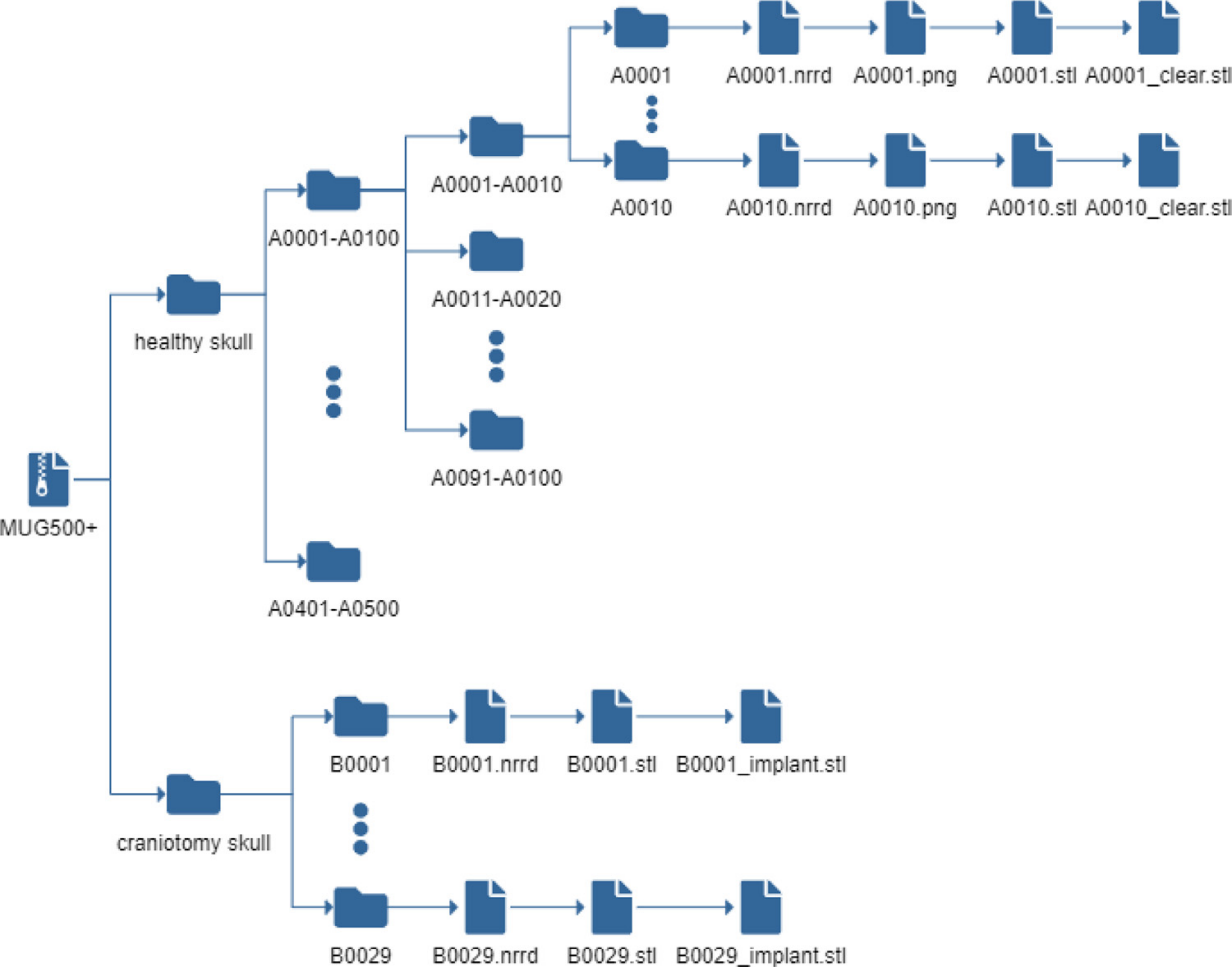
Folder structure of the *MUG500+* dataset.

Figure 1 shows the folder structure of the *MUG500+* dataset, which contains two types of skulls: the 500 healthy skulls and the 29 defective skulls from craniotomy. The folders of healthy skulls are named from *A0001* to *A0500*. Under each folder, the nearly raw raster data (*.nrrd*) file is the image data (size: 512×512×Z, Z is the number of axial slices) of the skull and the stereolithography (*.stl*) file is the corresponding mesh of the skull. The *.png* file, as a quick preview, shows a screenshot of the 3D skull model. The difference between *Axxxx.stl* and *Axxxx_clear.stl* is that, in *Axxxx_clear.stl*, most of the (background) noise, artefacts and structures that do not belong to the skull anatomically (e.g., the head spine) are removed.

**Fig. 2:**
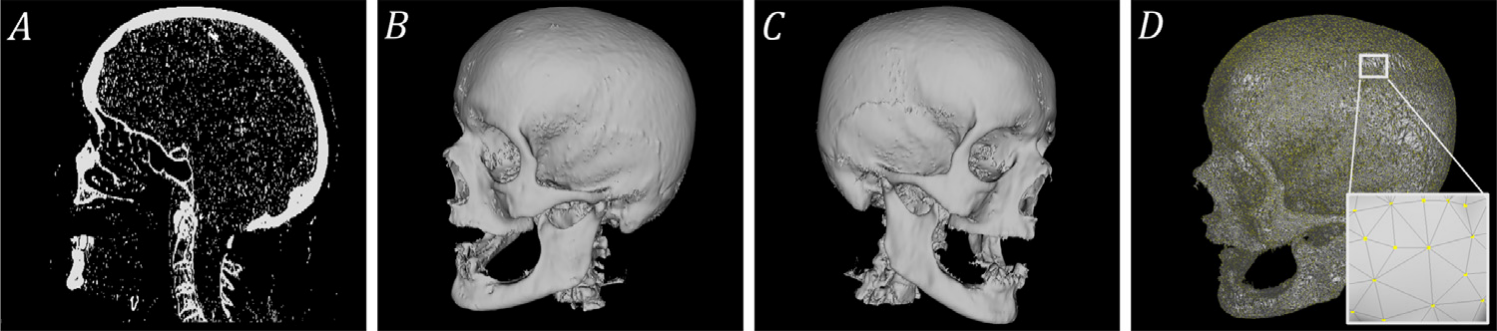
Illustration of a healthy skull *A0285.nrrd* in sagittal (A) and 3D (B,C) views. D: a 3D illustration of *A0285.stl*.

**Fig. 3:**
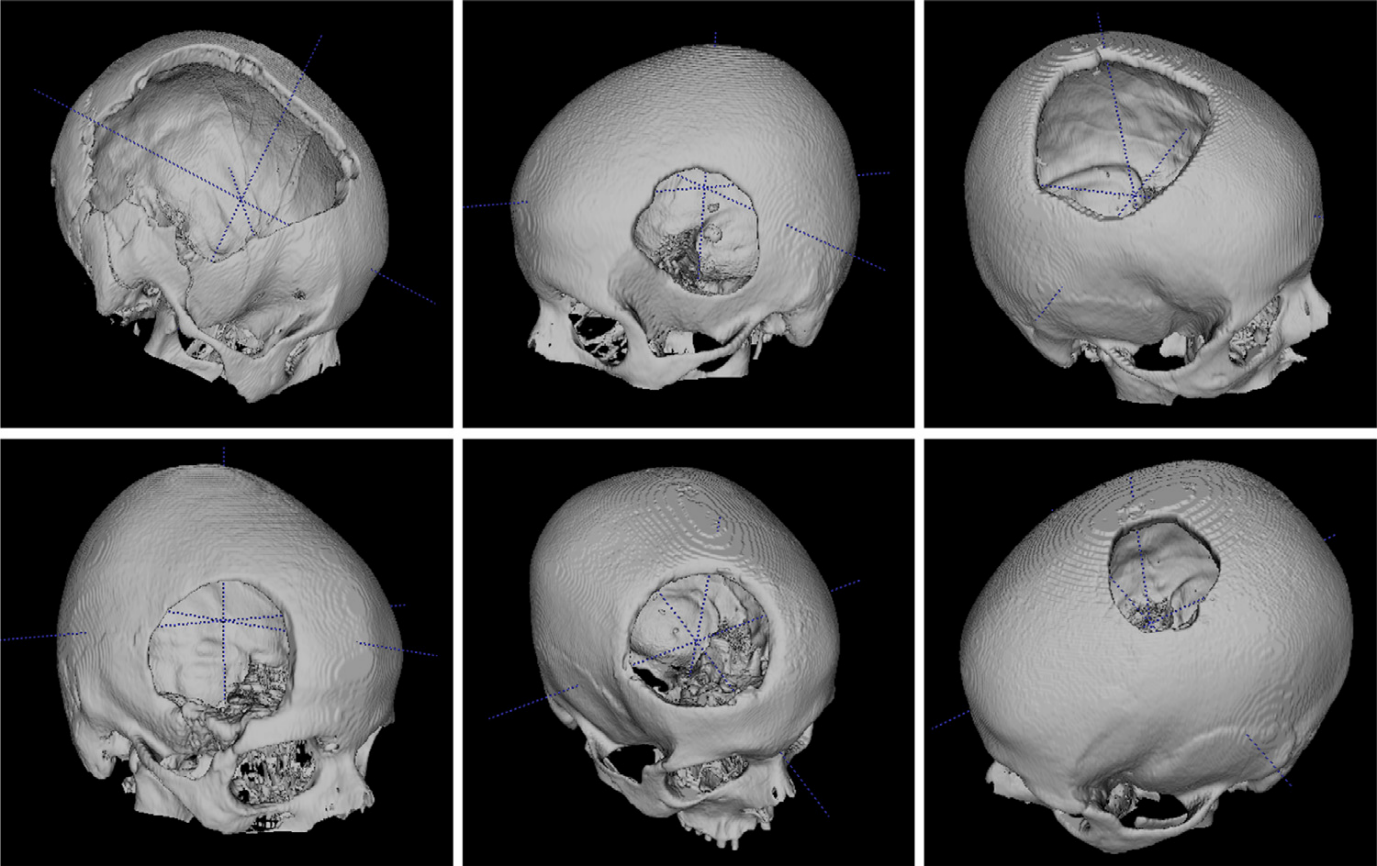
Illustration of craniotomy skulls with defects of various sizes, shapes and positions. The dataset could serve as an evaluation set for cranial implant design algorithms.

The folder of the craniotomy skulls are named from *B0001* to *B0029*. Under each folder, the *.nrrd* file is the image data (size: 512×512×Z) of the defective skulls. The *.stl* files are the meshes of the skull and the manually designed cranial implant. Figs. 2 and 3 show a healthy skull (*A0285*) and six craniotomy skulls (*B0001, B0002, B0004, B0006, B0016* and *B0019*) with various defects, respectively. Table 1 shows the meta information (resolution, slice thickness, etc) of the healthy and craniotomy skulls.

**Table 1:** Image information of the healthy and craniotomy skulls.

Image Information	Healthy skull	Craniotomy skull
Patients’ age (min/median/average/max)	18/63/61/119	-
Percentage of female patients	40%	-
x/y resolution	512×512	512×512
Number of axial slices (min/median/max)	-	147/167/291
Slice thickness	1.5 mm	0.5 mm

## 2 Experimental Design, Materials and Methods

Having a uniform collection of medical datasets and a standard operating procedure (SOP) for data processing not only makes the research outcome based on these datasets more reliable but also facilitate reproducibility of the results by other institutions, which is increasingly important nowadays. The *MUG500+* database was constructed based on the head CT scans acquired from the Medical University of Graz (MUG) in clinical routines. The head CT scans are originally in the format of Digital Imaging and Communications in Medicine (DICOM). For privacy considerations, a pseudonymization process, where the patients’ personal information such as age and gender were removed. For a high level overview of the dataset, Table 1 only provides the statistics (min, max, etc) of the patients’ age and gender distribution. The DICOM files are further converted into the *.nrrd* format, as is in the *MUG500+* database.

### 2.1 Skull generation from head CT scans

Both the healthy skulls (*.nrrd*) and craniotomy skulls (*.nrrd*) are segmented from head CT scans by medical experts based on a thresholding technique using 3D Slicer (https://www.slicer.org/) [9]. For each head CT, the segmentation threshold is decided specifically by the expert so that the complete cranial and facial bones on the skull can be preserved. The mesh files (*.stl*) of the skulls are extracted from the corresponding segmentation masks.

### 2.2 Computer-aided cranial implant design for the 29 craniotomy skulls

**Fig. 4:**
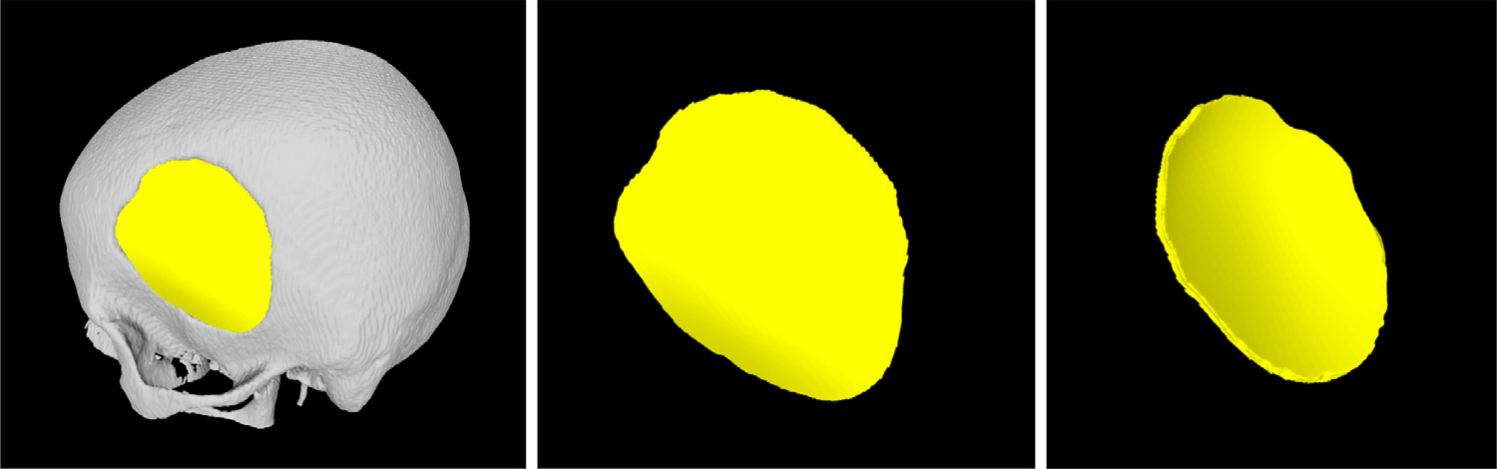
An illustration of a defective skull (*B0002.stl*) and the corresponding manually designed cranial implant (B0002_implant.stl).

The cranial implants of the 29 craniotomy skulls are designed by an expert using the Geomagic Sculpt software. The software takes as input the *.stl* version of the craniotomy skulls and the resulting implants can be exported in the same format (*.stl*). Fig. 4 shows an illustration of a craniotomy skull (in *gray*) with the corresponding cranial implant (in *yellow*). We have also recorded a tutorial video about the semi-automatic cranial implant design workflow with Geomagic Sculpt, which can be viewed at https://www.youtube.com/watch?v=FzaR3ydjaSc.

## Ethics Statement

This investigation was approved by the internal review board (IRB) of the Medical University of Graz, Austria (IRB: EK-32-177 ex 19/20).

## CRediT authorship contribution statement

**Jianning Li:** Data curation, Writing – original draft. **Marcell Krall:** Data curation. **Florian Trummer:** Data curation. **Afaque Rafique Memon:** . **Antonio Pepe:** Writing – original draft. **Christina Gsaxner:** Writing – original draft. **Yuan Jin:** . **Xiaojun Chen:** . **Hannes Deutschmann:** Data curation. **Ulrike Zefferer:** Supervision. **Ute Schäfer:** Supervision. **Gord von Campe:** Data curation, Supervision. **Jan Egger:** Data curation, Writing – original draft, Supervision.

## Declaration of Competing Interest

The authors declare that they have no known competing financial interests or personal relationships that could have appeared to influence the work reported in this paper.

## Footnotes

1 Note: We only uploaded the first 20 cases of the healthy skulls and the first 15 cases of the craniotomy skulls with implants, because there is a space limit on Figshare for private repositories and we did not want to make it public yet in case the reviewers suggest changes (note, once public, a repository on Figshare cannot be changed/removed by the authors anymore).
